# Interpersonal Trust across Six Asia-Pacific Countries: Testing and Extending the ‘High Trust Society’ and ‘Low Trust Society’ Theory

**DOI:** 10.1371/journal.pone.0095555

**Published:** 2014-04-23

**Authors:** Paul R. Ward, Loreen Mamerow, Samantha B. Meyer

**Affiliations:** 1 Discipline of Public Health, Flinders University, Adelaide, South Australia, Australia; 2 School of Public Health and Health Systems, University of Waterloo, Waterloo, Ontario, Canada; State University of New York, Oswego, United States of America

## Abstract

**Background:**

Trust is regarded as a necessary component for the smooth running of society, although societal and political modernising processes have been linked to an increase in mistrust, potentially signalling social and economic problems. Fukuyama developed the notion of ‘high trust’ and ‘low trust’ societies, as a way of understanding trust within different societies. The purpose of this paper is to empirically test and extend Fukuyama’s theory utilising data on interpersonal trust in Taiwan, Hong Kong, South Korea, Japan, Australia and Thailand. This paper focuses on trust in family, neighbours, strangers, foreigners and people with a different religion.

**Methods:**

Cross-sectional surveys were undertaken in 2009–10, with an overall sample of 6331. Analyses of differences in overall levels of trust between countries were undertaken using Chi square analyses. Multivariate binomial logistic regression analysis was undertaken to identify socio-demographic predictors of trust in each country.

**Results:**

Our data indicate a tripartite trust model: ‘high trust’ in Australia and Hong Kong; ‘medium trust’ in Japan and Taiwan; and ‘low trust’ in South Korea and Thailand. Trust in family and neighbours were very high across all countries, although trust in people with a different religion, trust in strangers and trust in foreigners varied considerably between countries. The regression models found a consistent group of subpopulations with low trust across the countries: people on low incomes, younger people and people with poor self-rated health. The results were conflicting for gender: females had lower trust in Thailand and Hong Kong, although in Australia, males had lower trust in strangers, whereas females had lower trust in foreigners.

**Conclusion:**

This paper identifies high, medium and low trust societies, in addition to high and low trusting population subgroups. Our analyses extend the seminal work of Fukuyama, providing both corroboration and refutation for his theory.

## Background

There have been a large number of scholars who have attempted to define, conceptualise and operationalise trust within the disciplines of sociology [1], [2], [3], [4], [5], political science [6], [7], and philosophy [8], [9], [10]. There is also scholarship on trust in other academic disciplines such as economics, social policy and management, but they are outside of the realm of this paper. Within sociology, there exist a number of useful conceptual reviews of trust [11], [12], [13], [14], [15] which reduce the need to provide a full and complete review here. A generally accepted definition of trust is ‘the mutual confidence that no party to an exchange will exploit the other’s vulnerability’ [16], with a trustworthy person having both good intentions and reasonable competence [9]. Nevertheless, we provide a brief overview in order to set the context and conceptual framework for this paper.

Trust is regarded as a necessary component for the smooth running of society [17] and the glue that holds society together [1]. Fukuyama [6] uses economic theory to argue for the necessity of trust, “it is very difficult to conceive of modern economic life in the absence of a minimum level of informal trust” (p.151). Without trust, individuals would be constantly trying to make complex decisions on how to live their lives, deciding on a case-by-case basis whether to trust their neighbour to look after their house whilst they are on holiday, a teacher to look after their children at school or a stranger they talk to at the bus stop to be courteous and not malevolent. These decisions and the time and energy taken to make them, are known as externalities in economic theory [6] which increase the efficiency of the system, be it a social system or economic system. However, there is a concern with the decline in trust in several democratic countries [7] and evidence suggests that modern social developments have led to the erosion of trust in these countries.

Luhmann argues that the act of trusting (or indeed not trusting) reduces complexity in society, allowing individuals to get on with other aspects in their lives [1]. Therefore, understanding the nature and extent of trust in society becomes extremely important – if we can understand which groups are distrusted or which groups are more likely to distrust others, we can begin to develop social policy and maybe social marketing to reduce the distrust, and in Giddens’ view, make society more smooth functioning.

Giddens’ argues that the ‘consequences of modernity’ have been profound on the nature and extent of trust [17] – people have become reflexive actors who use available information and also personal experience in order to make decisions about whether or not to trust another person [3]. Govier [9] similarly argues this when she says “Trust is not faith; we need not trust blindly” (p.7). This reflexivity or questioning of trust has led some researchers to suggest that Western society exhibits a generalized manifestation of distrust, akin to conspiracy theory [18]. This also reflects Hobbesian ideas that human relationships are built on suspicion, greed and competition, which lead to a default position of distrust. However, Govier [9] argues philosophically for trust to be the default position, although she also recognises that ‘blind trust’ can lead to complacency, “the fact that things “seem all right” or that we “have a good feeling about that person” is simply not an adequate basis for trust” (p.9). Govier cites Ghandi’s philosophy which was based on a person being ‘trustworthy unless they proved otherwise’ – if a stranger has not shown themselves to be untrustworthy, we should trust them.

The notion of the ‘default position of trust’ is critical to this paper, since we analyse trust in people that are known (e.g. family, neighbours) but also people that are unknown (e.g. strangers, foreigners) to our respondents. Trust in strangers cannot necessarily be built on ‘rational’ evidence or prior experience like Giddens suggests, unless an individual has had very bad experiences with lots of strangers. Therefore, for a person to decide whether or not to trust a stranger or foreigner, there must be some social or cultural norms underpinning the decision, often based on a constructed characterisation or stylised notion of them [9]. Indeed, Fukuyama [6] argues that “trust arises when a community shares a set of moral values in such a way as to create expectations of regular and honest behaviour” (p.153).

The work of Fukuyama is extremely important within this paper, since he brings culture and social norms into the mix. Fukuyama argues for the centrality of trust in economic development, along with sociologists who argue for its centrality in social development. He argues that the family and civil society create durable social institutions which are resilient to legislative and economic change – trust becomes the glue in these forms of social order. Fukuyama makes this abundantly clear when he says “If the institutions of democracy and capitalism are to work properly, they much coexist within certain premodern cultural habits that ensure their proper functioning” (p.11). He goes on to say “Law, contract, and economic rationality and prosperity…. must as well be leavened with reciprocity, moral obligation, duty toward community, and trust…. The latter are not anachronisms in a modern society but rather the sine qua non of the latter’s success” (p.11). Given this centrality of trust, he then attempts to understand why some societies have lower or higher trust.

Fukuyama develops a hypothesis about ‘low trust societies’ and ‘high trust societies’ which is interrogated within this paper. He argues that within low trust societies, social relations/connections are primarily within the family – familial piety – and that these societies will be less trusting of people outside of the family [6]. His work examines in great depth both Confucianist and non-Confucianist countries in order to develop and argue his thesis. In terms of social networks or social capital, Fukuyama argues that “communities with the strongest internal ties will have the weakest bonds with the outside” (p.154), and he links the internal ties to higher trust within groups and lower trust outside of those groups. In an Asian context, he links trust in the family, as the most important social unit, to Confucianism which regards the family as the most important social unit. He provides extensive evidence of the ways in which the family is still the central unit in Confucianist societies and of the impact this has on reduced trust in people and institutions outside the family. However, he argues that in having less trust outside the family, people are not necessarily individualistic (in an economic rationalist sense), but view the family as the central and most important social unit. Nevertheless, Fukuyama argues that “the lack of trust outside the family makes it hard for unrelated people to form groups or organizations” (p.73).

Fukuyama shows how businesses have developed as family owned/run organisations more in Chinese-centred countries like Taiwan and Hong Kong, but less so in ‘less Chinese’ countries like Japan – with South Korea having parts of both Confucianist philosophy but also a more open attitude to trusting others due to State interventions in attempts at ‘globalising’ South Korea. Essentially, Fukuyama presents the case for Taiwan, Hong Kong and to a slightly lesser degree South Korea being ‘low trust societies’ – having high trust in the family, but low trust outside of the family. He presents the case for Japan being a high trust society, alongside countries like the US, the UK and Germany. Whilst he does not explicitly talk about trust in Australia, we would suggest that it is closer to the US and UK, and thus may be hypothesised as a high trust society. In this paper we also present data on interpersonal trust in Thailand, although Fukuyama does not specifically focus on it. He mentions (p.71) that the small enclaves of Chinese people and culture in Thailand may make it similar to the Chinese influenced countries like Hong Kong and Taiwan, although he also shows that Thailand, like Singapore and Malaysia, accepts and encourages foreign investment, thereby reducing the focus on the family, and potentially increasing trust.

There have been theoretical [19], [20] and empirical [21], [22], [23] critiques of Fukuyama’s work on trust. The theoretical critiques tend to focus on the possibility of being able to have generalised notions of trust, as opposed to context-dependent and locally situated definitions. The empirical critiques have tended to examine the levels and predictors of trust between two countries. For example, Yamagishi & Yamagishi [23] explored the differences in trust between Japan and America, finding some similarities to Fukuyama – trust was generally higher in America than Japan. However, they found different social relations, with Japanese having more durable exchange relations than Americans. Further research comparing Japan and America found little support for differences in trust on the basis of cultural differences between the countries. [21] However, these studies only compared Japan and America, both of which according to Fukuyama are high trust societies, partly due to them not being based on Confuciansim. Therefore, further research is required to examine social trust between Confucianist and non-Confucianist societies.

Fukuyama’s seminal work sets up a number of hypotheses which we test within this paper. The first major hypothesis relates to there being higher trust in non-Confucianist societies as compared to Confucianist societies. The second major hypothesis relates to trust in families being higher in Confucianist societies as compared to non-Confucianist societies. The third major hypothesis relates to trust in social groups other than families being lower in Confucianist socieites as compared to non-Confucianist societies. In order to empirically tests Fukuyama’s hypothesess, we present data on interpersonal trust (trust in individuals rather than individuals/systems) in Taiwan, Hong Kong, South Korea, Japan, Australia and Thailand. Corroboration of Fukuyama’s hypothesese would see Australia, Japan and South Korea (non-Confucianist societies) as ‘high trust societies’ and Taiwan and Hong Kong (Confucianist societies)as ‘low trust societies’. The ‘high trust’ societies would have lower trust in families and higher trust in non-family social groups. Given Fukuyama’s comments on Thailand, one may also regard it a ‘low trust society’, although this was not explicitly stated by Fukuyama.

In addition to empirically testing the above hypotheses, the paper extends Fukuyama’s work by identifying the population groups within each country who have higher or lower trust, thereby not assuming homogeneity within each country. Fukuyama’s work was seminal in providing a sociological analysis of *between-country* trust, but he did not examine *within-country* trust, which is understandable given the size of the task and the research aims of his work. In this way, our paper both operationalizes and extends the sociology of trust.

## Methods and Dataset

### Ethics Statement

Appropriate approvals were obtained within each country to undertake the individual surveys. The authors were granted ethics approval from Flinders University Social and Behavioural Research Ethics Committee to obtain and use the collected data for secondary analysis (project number 5221).

### Data Collection

The data presented in this paper come from a larger survey across six Asia-Pacific counties: Australia (Flinders University), Hong Kong (Chinese University Hong Kong), Japan (Chiba University), South Korea (Seoul National University), Taiwan (National Taiwan University), Thailand (King Prajadhipok Institute). The details and critique of the survey methods across the six countries have been published elsewhere [24], [25], although a brief synopsis is provided here for readers to understand the results presented in the paper. Details of method used within the study countries are also published elsewhere [26], [27], [28], [29], [30].

We were cognisant of the various methodological issues related to cross-country research, which could potentially lead to difficulties in interpreting the data. In order to respond to these issues, we undertook a number of strategies to make each country-specific survey as comparable as possible. Initially, we used questions from pre-validated questionnaires, including the World Values Survey [31] and the General Social Survey [32], to develop the questionnaire. The English version of the questionnaire was then validated [33] and subsequently translated into the language of the host country. Consultations were then undertaken with academics from each of the collaborating universities in order to further refine the questionnaires. Pilot studies were then undertaken in each country to validate the questionnaires and to make sure that respondents understood the questions and response categories, reducing the potential for systematic bias. Our decision to include the same questions and response categories across all countries (albeit in different languages) fits in with recognised standards for cross-country survey research [34], [35] Nevertheless, given that trust is both socially and culturally mediated, a potential limitation of the study may reside in slightly different interpretations of ‘trust’ both within and between countries. In addition, research has identified that respondents in different countries may interpret response categories slightly differently [36]. The potential for slightly different interpretations of both the question and the response categories has been termed ‘living with imperfect comparisons’ [37], which remains a potential limitation for all cross-country comparative research.

Data were collected in each country between 2009 and 2010, using either face-to-face or postal survey techniques. The total sample size was 6331∶681 in Hong Kong; approximately 1000 in Australia and Japan; and 1200 in each of South Korea, Taiwan and Thailand.

This paper presents an analysis of questions on inter-personal trust. The question appeared in the questionnaire as: How much do you **trust** various **groups of people**? (emphasis in original). There were four response categories: Trust them completely; Trust them a little; do not trust them very much; do not trust them at all. The survey provided a list of 11 different groups against which respondents were asked to rate their level of trust. For the purpose of this paper, we focus on trust in five groups: family, neighbours, strangers, people with a different religion and foreigners. We have chosen family and neighbours because they represent groups ‘known’ to the respondents, whereas the other groups are ‘unknown’. This allows us to examine trust on the basis of family ties and in-depth knowledge in addition to trust without these bases, thereby allowing us to further examine Fukuyama’s hypothesis across six countries. The questionnaire also included 11 questions on socio-demographics such as income, gender and age.

## Data Analysis

After the surveys had been undertaken, merging and cleaning of the dataset was conducted by academics at Seoul National University. In addition, once we had a cleaned SPSS dataset, data were weighted for each country on the basis of age and sex, to mitigate potential bias in age-sex response rates. Responses to surveys were generally under-represented by males and younger respondents, and therefore the age-sex weighting created a nationally representative dataset for analysis.

To achieve suitable levels for analysis and a basis for comparison, responses to survey items enquiring about individual’s trust in various groups of people were dichotomised. Retaining the original four categories did not yield enough cases in each category to allow multivariate analysis across each country. The new dichotomised variable does not have the fine grained differences in trust as the original variable, and this may represent a potential limitation in interpreting the results of the paper. For all countries, ‘trust them completely’ and ‘trust them a little’ were combined to form a categorical variable labelled ‘medium/high trust’, whilst responses in the form of ‘do not trust them very much’ and ‘do not trust them at all’ were combined into ‘low/no trust’. Beyond purely descriptive statistics, we were also interested in whether (socio) demographic attributes of respondents hold predictive qualities for trust invested in different groups of people. For this purpose, a pool of categorical predictor variables was generated comprising respondents’ sex, age group, marital status, work status, household income, as well as subjective health status and whether respondents reported a chronic health condition.

Chi-Square analyses were conducted to examine the relationship between country and trust in various groups of individuals, including self-reported levels of trust in family, neighbours, strangers, foreigners, and people with a different religion. All chi-square analyses revealed a significant association between different countries and level of trust placed in the separate groups of individuals (all chi-square analyses significant at p<.001). In order to develop a better understanding of potential mechanisms driving these associations, we examined the standardised residuals obtained for each cell. This allowed us to identify cell counts, which made significant contributions to the overall significance of the particular association under investigation. To facilitate the interpretation of z-scores, the reader is reminded that z-scores with a magnitude equal to or higher than ±1.96 are associated with a two-tailed significance level of p = 0.05, z-scores of ±2.575 refer to a two-tailed probability of p = 0.01 and z-scores of ±3.3 are associated with two-tailed significance at p = 0.001.

For the regression analyses, the predicted level of the dichotomised outcome variables (i.e. level of trust) was specified in the direction of the majority of responses. For instance, for a group of people respondents indicated to trust more frequently than not, the regression model was specified to predict trust. An outcome variable for which more low/no trust responses were observed, low/no trust was predicted. This procedure does not change the analysis for it does not impact on the relationships between variables, but facilitates interpretation of the regression results. Data were analysed using the SPSS version 21.0 (SPSS Inc., Chicago, IL, USA). Binomial logistic regression models were used to investigate associations for all six countries (Hosmer & Lemeshow, 2000). All demographic predictor variables were entered into the analysis as categorical variables. Bivariate logistic regression analyses were performed to examine the relationship between the individual demographic predictors and the various recipients of trust. Only items showing an association at the p<0.25 level were entered into multiple binary logistic regression analyses (Hosmer and Lemeshow, 2000). Following suggestions by Field (2009), for the purposes of the present investigation the method of choice for conducting regression analyses was to enter relevant predictor variables in one block rather than stepwise procedures. Predictor variables that were entered into the model but returned as not significant were in turn tested against models containing only significant predictor variables. This process allowed for the comparison of several models, resulting in a final model containing only variables, which significantly contributed to the model fit. For each outcome variable, predictor variables included in the regression model were checked for multicollinearity. Given the relatively high number of regression analyses presented in this paper, there is a possibility of some spurious regression models, and therefore caution needs to be taken with p values close to 0.5.

## Results

### General Levels of Trust

Table 1 outlines the proportions of respondents indicating ‘trust a little’ or ‘trust a lot’ (called ‘higher trust’ throughout the paper) in each of the countries. The general trend in Australia was for respondents to report higher levels of trust than in other countries. Across all categories of groups and individuals, 81% of respondents indicated higher trust. Family was the group which respondents indicated a higher level of trust (95%), followed by neighbours (86%), foreigners (85%) and people with a different religion (84%). Only 57% of respondents indicated higher trust in strangers. Overall, we may categorise Australia as a ‘high trust society’.

**Table 1:** Proportion of respondents indicating higher trust in various groups or individuals.

	Family	Neighbours	Different religion	Foreigners	Strangers	Mean % for each country
Australia	94.8	86.0	84.3	85.2	56.7	81.4
Hong Kong	98.5	75.4	64.7	59.3	16.9	63.0
Japan	98.3	79.5	29.6	28.2	33.0	53.7
South Korea	99.1	79.2	37.6	24.3	16.6	51.4
Taiwan	98.8	77.3	61.7	43.7	16.7	59.6
Thailand	99.4	92.4	23.1	20.8	10.3	49.2
Mean % for each category of trust	98.2	81.6	50.2	43.6	25.0	

Hong Kong had the second highest levels of trust, with 63% of respondents indicating higher trust across the various groups and individuals. Family received higher trust by 99% of respondents, followed by neighbours (75%), people with a different religion (65%), and foreigners (59%). Strangers were the only group in Hong Kong with relatively low trust, with only 17% of respondents indicating higher trust in this group. In Taiwan, the overall level of trust across the groups and individuals was 60%, making it similar to Hong Kong. The group most frequently trusted in Taiwan was family (99%), followed by neighbours (77%) and people with a different religion (62%). Trust was much lower for foreigners (44%) and even lower for strangers (17%). Other than trust in foreigners, which was higher in Hong Kong than Taiwan (50% and 44% respectively), Hong Kong and Taiwan had similar levels of trust across the groups and individuals, and relative to other countries, we may therefore categorise them as ‘medium level trust societies’, a categorization not identified in Fukuyama’s thesis.

Trust in Japan was lower than the previous three countries (54%). Whereas family (98%) and neighbours (80%) were highly trusted, lower trust was found in people with a different religion (30%) and foreigners (28%). However, trust in strangers (33%) was higher than in either Hong Kong or Taiwan, which both had 17% of respondents indicating higher trust. In South Korea a pattern similar to that observed for Japan was found, and a similar overall level of trust across the individuals and groups (51%). There were high levels of trust in family (99%) and neighbours (79%), but lower levels of trust in people with a different religion (38%), foreigners (24%) and strangers (17%). Again, trust in strangers is the outlier here, with the very low levels of trust in South Korea being similar to Hong Kong and Taiwan, but much lower than Japan. Thailand had the lowest overall level of trust across the individuals and groups, with 49% of respondents indicating higher trust, making it similar to Japan and South Korea (54% and 51% respectively). As with all other countries, trust was highest for family (99%) followed by neighbours (92%). However, there was much less trust in people with a different religion (23%), foreigners (21%) and strangers (10%). Compared to Japan and South Korea, Thailand had higher trust in neighbours, but much lower trust in people with a different religion and strangers. Notwithstanding these differences, we categorise Japan, South Korea and Thailand as ‘lower trust societies’.

In order to further analyse the similarities and differences outlined above, Table 2 presents the Chi-square output and z-scores for each of the countries across each of the individuals and groups. In terms of trust in neighbours (χ^2^ (5, 5941) = 145.1, p<.001), Australia and Thailand had significantly fewer respondents indicating low trust (z = −2.8 and z = −8.4, respectively), whereas in all other countries z-scores with a magnitude of 2.0 or over were observed, indicating higher numbers of respondents having lower trust in neighbours. Overall, Australia and Thailand had lower levels of trust in neighbours significantly less frequently than the other countries.

**Table 2:** Chi-square and z-scores for high and low trust across the countries.

		Chi-square	AU	HK	JPN	SK	TH	TWN
**Neighbours**		?^2^ (5, 5941) = 145.1, p<.001						
	*Low trust* *		**−2.8**	**4.1**	**2.0**	**2.2**	**−8.4**	**4.0**
	*Medium/High* **		1.3	−1.9	−0.9	−1.0	**3.9**	−1.8
**Strangers**		?^2^ (5, 5845) = 747.51, p<.001						
	*Low trust* *		**−11.0**	**2.1**	**−3.2**	**2.6**	**5.4**	**2.9**
	*Medium/High* **		**19.5**	**−3.8**	**5.8**	**−4.7**	**−9.6**	**−5.1**
**Foreigners**		?^2^ (5, 5424) = 1102.40, p<.001						
	*Low trust* *		**−16.4**	**−6.0**	**5.2**	**6.3**	**9.0**	−1.1
	*Medium/High* **		**19.6**	**7.1**	**−6.3**	**−7.5**	**−10.8**	1.4
**Different religion**		?^2^ (5, 5406) = 1014.65, p<.001						
	*Low trust* *		**−14.1**	**−6.0**	**7.7**	**3.9**	**11.5**	**−6.3**
	*Medium/High* **		**14.9**	**6.4**	**−8.1**	**−4.1**	**−12.1**	**6.6**

*Response options 'Do not trust them at all' and 'Do not trust very much'.

**Response options 'Trust them a little' and 'trust them completely'.

Examining z-scores for the associations for medium/high trust in strangers (χ^2^ (5, 5845) = 747.51, p<.001), cell counts were significantly higher than expected in Australia (z = 19.5) and Japan (z = 5.8), but significantly lower than expected in Hong Kong (z = −3.8), South Korea (z = −4.7), Taiwan (z = −5.1) and Thailand (z = −9.6). In other words, Australia and Japan had higher levels of trust in strangers significantly more frequently than the other countries.

In terms of trust in foreigners (χ^2^ (5, 5424) = 1102.40, p<.001), significantly higher than expected cell counts for medium/high level of trust in foreigners were observed for Australia (z = 19.6) and Hong Kong (z = 7.1), whilst significantly lower than expected cell counts were obtained for Japan (z = −6.3), South Korea (z = −7.5) and Thailand (z = −10.8). Observed cell counts in Taiwan did not contribute to the association. Therefore, Australia and Hong Kong had higher levels of trust in foreigners significantly more frequently than the other countries.

For the associations for trust in people with a different religion (χ^2^ (5, 5406) = 1014.65, p<.001), cell counts were for medium/high trust were significantly higher than expected for Australia (z = 15.9), Hong Kong (z = 6.4) and Taiwan (z = 6.6), whereas for Japan (z = −8.1), South Korea (z = −4.1) and Thailand (z = −12.1) had significantly lower than expected observed cell frequencies. These analyses show that Australia, Hong Kong and Taiwan had higher levels of trust in people with a different religion significantly more frequently than the other countries.

### Trust within Countries

The rest of the paper focuses on the regression models which examine the main sub-populations with either low or high trust in each country. This analysis extends the work of Fukuyama and facilitates appropriate policy responses to increase trust within particular groups in each country. Given the overwhelmingly high levels of trust in the family, it was not possible to generate regression models due to the lack of variability. Therefore, we only present data on trust in neighbours, people of a different religion, strangers and foreigners. Within the following sections, we use the term higher trust to refer to people who responded to either ‘trust them completely’ or ‘trust them a little’ and the term lower trust to refer to people who responded to either ‘do not trust them very much’ or ‘do not trust them at all’.

### Trust in Neighbours

#### Australia

Overall, 86% of respondents had higher trust in neighbours. Four independent variables were associated with trust in neighbours: age, income, work status and subjective health (see Table 3). Younger people were much less likely to trust neighbours than older people (aged 60 years or over), with people aged less than 20 years being over 99% less likely to trust (OR 0.01, 95% CI 0.00–0.05) and people aged 20–29 and 30–39 being less than 90% likely to trust neighbours (OR 0.06, 95% CI 0.02–0.25; and OR 0.08, 95% CI 0.02–0.31 respectively). Compared to people working full-time, part-time employed people were over 5 times more likely to trust neighbours (OR 5.39, 95% CI 2.16–13.47) and people working without pay were over 2.5 times more likely to trust neighbours (OR 2.62, 95% CI 1.14–6.03). Income was strongly associated with trust in neighbours, with people on higher incomes having higher trust. Compared to people earning less than $30,000, people earning either $60,000-$89,999 or $90,000-$119,999 were around 3 times more likely to trust neighbours (0R 2.87, 95% CI 1.20–6.88, and OR 3.14, 95% CI 1.12–8.82 respectively) and people earning $120,000-$149,999 were over 6 times more likely to trust (OR 6.20, 95% CI 1.79–21.45). People with worse subjective health were approximately 50% less likely to trust neighbours than people with better subjective health (OR 0.47, 95% CI 0.28–0.79).

**Table 3:** Regression model for trust in neighbours in Australia.

Predictor	OR (95% CI)	P
**Age (≥60)**		**<0.001**
<20	0.009 (0.001–0.053)	<0.001
20–29	0.061 (0.015–0.253)	<0.001
30–39	0.076 (0.018–0.312)	<0.001
40–49	0.235 (0.052–1.058)	0.059
50–59	0.179 (0.046–0.699)	0.013
**Work status (Full time/self-employed)**		**0.002**
Part time	5.390 (2.157–13.469)	<0.001
Working without pay/student/unemployed	2.618 (1.137–6.028)	0.024
Retired/pensioner	1.145 (0.296–4.422)	0.845
Household duties	2.405 (0.871–6.635)	0.090
**Monthly household income (<AUS$30,000)**		**0.007**
AUS$30,000–59,999	1.444 (0.629–3.317)	0.386
AUS$60,000–89,999	2.870 (1.197–6.878)	0.018
AUS$90,000–119,999	3.142 (1.119–8.824)	0.030
AUS$120,000–149,999	6.196 (1.790–21.452)	0.004
≥ AUS$150,000	4.456 (1.448–13.711)	0.009
**Subjective health status (Very good, good)**		**0.005**
Fair, bad, very bad	0.469 (0.278–0.791)	0.005

#### Hong Kong

All predictors were entered into the regression model but it was not significant and therefore no model is presented here.

#### Japan

In Japan, 79.5% of respondents had higher trust in neighbours. Four independent variables were associated with trust in neighbours: age, income, marital status and subjective health (see Table 4). Compared to individuals aged 60 years and above, all other (younger) age groups were significantly less likely to indicate higher trust in neighbours. Indeed, as age group gets younger, trust in neighbours becomes lower in a step-wise fashion, with people aged 50–59 being 59% less likely to trust (OR 0.41, 95% CI 0.24–0.72), people aged 40–49 being 63% less likely to trust (OR 0.37, 95% CI 0.21–0.67), people aged 30–39 being 73% less likely to trust (OR 0.23, 95% CI 0.13–0.39) and people aged 20–29 being 87% less likely to trust neighbours (OR 0.13, 95% CI 0.07–0.24). Compared to married/cohabiting respondents, people who were separated, divorced or widowed were almost 50% less likely to report higher trust in neighbours (OR 0.51, 95% CI 0.30–0.87). In terms of income, people in the highest income group were over 70% more likely to have higher trust in neighbours than people in the lowest income group (OR 1.71, 95% CI 1.04–2.81). People with worse subjective health were approximately 40% less likely to trust neighbours than people with better subjective health (OR 0.63, 95% CI 0.43–0.90).

**Table 4:** Regression model for trust in neighbours in Japan.

Predictor	OR (95% CI)	P
**Age (≥60)**		**<0.001**
20–29	0.125 (0.066–0.238)	<0.001
30–39	0.228 (0.132–0.393)	<0.001
40–49	0.371 (0.207–0.665)	0.001
50–59	0.413 (0.238–0.718)	0.002
**Marital status (Married/cohabitating)**		**0.040**
Separated/divorced/widowed	0.513 (0.301–0.874)	0.014
Never married	1.129 (0.648–1.966)	0.668
**Annual household income (Lower third)**		**0.049**
Middle third	0.951 (0.640–1.412)	0.802
Upper third	1.708 (1.036–2.814)	0.036
**Subjective health status (Very good, good)**		**0.012**
Fair, bad, very bad	0.625 (0.434–0.900)	0.012

#### South Korea

In South Korea, 79% reported higher trust in neighbours. Two independent variables were associated with trust in neighbours: age and subjective health (see Table 5). Respondents under the age of 20, between 20–29 and between 30–39 years were significantly less likely to have higher trust in neighbours than people aged 60 years or over (OR 0.13, 95% CI 0.06–0.30; OR 0.30, 95% CI 0.17–0.51; OR 0.53, 95% CI 0.31–0.91 respectively). People with worse subjective health were almost 50% less likely to trust neighbours than people with better subjective health (OR 0.51, 95% CI 0.37–0.72).

**Table 5:** Regression model for trust in neighbours in South Korea.

Predictor	OR (95% CI)	P
**Age (≥60)**		**<0.001**
<20	0.131 (0.058–0.299)	<0.001
20–29	0.298 (0.174–0.511)	<0.001
30–39	0.528 (0.307–0.910)	0.021
40–49	0.753 (0.429–1.324)	0.325
50–59	0.945 (0.496–1.801)	0.864
**Subjective health status (Very good, good)**		**<0.001**
Fair, bad, very bad	0.514 (0.369–0.717)	<0.001

#### Taiwan

In Taiwan, 77% of respondents reported high trust in neighbours. Three independent variables were associated with trust in neighbours: work status, marital status and income (see Table 6). Compared to married or cohabitating, respondents who were separated/divorced/widowed or never married were around 50% less likely to report higher trust in neighbours (OR 0.55, 95% CI 0.33–0.93; OR 0.44, 95% CI 0.31–0.63 respectively). Compared to people reporting full-time employment, people in part-time employment were 50% less likely have higher trust in neighbours (OR 0.49, 95% CI 0.28–0.86) and retired people were twice as likely to report higher trust (OR 2.02, 95% CI 1.05–3.89). In terms of income, people in the highest income group were over 50% more likely to have higher trust in neighbours than people in the lowest income group (OR 1.52, 95% CI 1.04–2.22).

**Table 6:** Regression model for trust in neighbours in Taiwan.

Predictor	OR (95% CI)	P
**Work status (Full time/self-employed)**		**0.005**
Part time	0.486 (0.276–0.859)	0.013
Work without pay/student/unemployed/other	1.139 (0.585–2.215)	0.702
Retired/pensioner	2.017 (1.046–3.889)	0.036
Household duties	0.674 (0.389–1.168)	0.160
**Marital status (Married/cohabitating)**		**<0.001**
Separated/divorced/widowed	0.553 (0.330–0.927)	0.025
Never married	0.438 (0.305–0.630)	<0.001
**Monthly household income (Lower third)**		**0.062**
Middle third	1.432 (0.954–2.149)	0.083
Upper third	1.515 (1.036–2.217)	0.032

#### Thailand

In Thailand, 92.4% of respondents had higher trust in neighbours. Only one independent variable was included in the regression model, namely subjective health status (see Table 7). People with worse subjective health were almost 50% less likely to trust neighbours than people with better subjective health (OR 0.49, 95% CI 0.32–0.76).

**Table 7:** Regression model for trust in neighbours in Thailand.

Predictor	OR (95% CI)	P
**Subjective health status (Very good, good)**		**0.001**
Fair, bad, very bad	0.492 (0.320–0.756)	0.001

### Trust in Strangers

#### Australia

In Australia, 57% of respondents had higher trust in strangers. Two independent associations were found in the regression model, namely age and sex (see Table 8). Younger people are much less likely to trust strangers than older people (60 years or older), with people aged less than 20 years being 95% less likely to trust strangers (OR 0.05, 95% CI 0.02–0.14). Females were almost 60% more likely to trust strangers than males (OR 1.57, 95% CI 1.14–2.17).

**Table 8:** Regression model for trust in strangers in Australia.

Predictor	OR (95% CI)	P
**Age (≥60)**		**<0.001**
<20	0.048 (0.017–0.136)	<0.001
20–29	0.088 (0.050–0.154)	<0.001
30–39	0.285 (0.169–0.480)	<0.001
40–49	0.311 (0.184–0.525)	<0.001
50–59	0.469 (0.271–0.812)	0.007
**Sex (Male)**		**0.006**
Female	1.571 (1.140–2.166)	0.006

#### Hong Kong

In Hong Kong, only 17% of respondents had higher trust in strangers, and therefore the regression model was based on lower trust since this included the majority of variance. Two independent variables were included in the model: sex and work status (see Table 9). Females were over 4 times more likely to have lower trust in strangers than males (OR 4.28, 95% CI 2.59–7.07). People who were retired were over 75% less likely to report lower trust than full-time employed people, indicating a higher level of trust by this, normally older, group (OR 0.24, 95% CI 0.14–0.41).

**Table 9:** Regression model for distrust in strangers in Hong Kong.

Predictor	OR (95% CI)	P
**Sex (Male)**		**<0.001**
Female	4.283 (2.593–7.073)	<0.001
**Work status (Full time/self-employed)**		**<0.001**
Part time	1.225 (0.434–3.463)	0.701
Work without pay/student/unemployed/other	0.806 (0.438–1.482)	0.487
Retired/pensioner	0.239 (0.141–0.406)	<0.001
Household duties	1.077 (0.375–3.091)	0.890

#### Japan

In Japan, 33% of respondents had higher trust in strangers, and therefore the regression model was based on lower trust since this represents 67% of respondents. There were two independent associations in the regression model; age and subjective health status (see Table 10). All age groups under 60 years were more likely to have lower trust than people aged 60 years or above. For example, people aged 20–29 were almost 4 times more likely to have lower trust in stranger (OR 3.81, 95% CI 2.39–6.08) and people aged 30–29 years were over 3.5 times more likely to have lower trust (OR 3.66, 95% CI 2.37–5.65). People with poor subjective health were 70% more likely to report lower trust in strangers than people with better subjective health (OR 1.70, 95% CI 1.27–2.28).

**Table 10:** Regression model for distrust in strangers in Japan.

Predictor	OR (95% CI)	P
**Age (≥60)**		**<0.001**
20–29	3.812 (2.391–6.079)	<0.001
30–39	3.655 (2.365–5.647)	<0.001
40–49	2.439 (1.593–3.735)	<0.001
50–59	1.554 (1.066–2.263)	0.022
**Subjective health status (Very good, good)**		**<0.001**
Fair, bad, very bad	1.701 (1.271–2.275)	<0.001

#### South Korea

In South Korea, only 17% of respondents had higher trust in strangers. In the regression model (based on lower trust), there were two independent associations; income and chronic condition (see Table 11). Respondents in the middle third of monthly household income groups were twice as likely to have lower trust in strangers compared to those in the upper third (OR 2.053, 95% CI 1.23–3.43), but it was not statistically significant for the lowest income group. Respondents with a chronic condition were almost 4 times as likely to have lower trust in strangers compared with individuals without a chronic condition (OR 3.85, 95% CI 0.96–15.37).

**Table 11:** Regression model for distrust in strangers in South Korea.

Predictor	OR (95% CI)	P
**Monthly household income (Upper third)**		**0.017**
Lower third	1.393 (0.941–2.061)	0.098
Middle third	2.053 (1.229–3.428)	0.006
**Chronic health condition (No)**		**0.056**
Yes	3.849 (0.964–15.372)	0.056

#### Taiwan

In Taiwan, only 17% of respondents had higher trust in strangers. The regression model (for lower trust) included only the variable on income (see Table 12). Respondents in the upper third income group were over 30% less likely to have lower trust in strangers compared to those in the lower third (OR 0.66, 95% CI 0.45–0.98).

**Table 12:** Regression model for distrust in strangers in Taiwan.

Predictor	OR (95% CI)	P
**Monthly household income (Lower third)**		**0.061**
Middle third	1.031 (0.653–1.630)	0.895
Upper third	0.662 (0.447–0.982)	0.040

#### Thailand

In Thailand, only 10% of respondents had higher trust in strangers, representing the lowest percentage of all countries in the study. The regression model only included one variable, age, and within this variable only two categories were statistically significant (see Table 13). Compared to individuals aged 60 years or over, respondents aged 50–59 years were almost 4 times more likely to have lower trust in strangers (OR 3.78, 95% CI 1.47–9.70).

**Table 13:** Regression model for distrust in strangers in Thailand.

Predictor	OR (95% CI)	P
**Age (≥60)**		**0.057**
<20	0.662 (0.284–1.542)	0.339
20–29	1.158 (0.638–2.103)	0.629
30–39	1.178 (0.650–2.135)	0.589
40–49	1.085 (0.596–1.976)	0.790
50–59	3.776 (1.470–9.697)	0.006

### Trust in Foreigners

#### Australia

In Australia, 85% of respondents had higher trust in foreigners. The regression model included five independent associations; sex, age, work status, income and subjective health status (see Table 14). Females were 80% more likely to trust foreigners than males (OR 1.80, 95% CI 1.00–3.24). Compared to respondents aged 60 or over, respondents under 20 years were 98% less likely to trust foreigners (OR 0.02, 95% CI 0.00–0.10) although no significant associations were found for other age groups. Compared to respondents in full-time employment, people in part-time employment as well as those retired were approximately 4 times more likely to have higher trust in foreigners (OR 4.05, 95% CI 1.51–10.89; and OR 3.74, 95% CI 1.17–11.89 respectively). Higher income was associated with significantly higher trust in foreigners. Compared to individuals with an income lower than $30,000, people on incomes of $120,000–149,999 and over $150,000 were around 5 times more likely to have higher trust in strangers (OR 4.70, 95% CI 1.17–18.86; and OR 5.01, 95% CI 1.34–18.80 respectively). Respondents with poorer subjective health were over 50% less likely to have higher trust than people with better perceived health (OR 0.45, 95% CI 0.26–0.80).

**Table 14:** Regression model for trust in foreigners in Australia.

Predictor	OR (95% CI)	P
**Sex (Male)**		**0.051**
Female	1.798 (0.997–3.241)	0.051
**Age (≥60)**		**<0.001**
<20	0.020 (0.004–0.104)	<0.001
20–29	0.658 (0.205–2.106)	0.480
30–39	0.456 (0.163–1.275)	0.134
40–49	1.539 (0.496–4.772)	0.456
50–59	1.078 (0.374–3.109)	0.890
**Work status (Full time/self-employed)**		**0.018**
Part time	4.048 (1.505–10.888)	0.006
Working without pay/student/unemployed/other	1.716 (0.619–4.757)	0.299
Retired/pensioner	3.736 (1.174–11.893)	0.026
Household duties	3.274 (0.848–12.635)	0.085
**Monthly household income (<AUS$30,000)**		**0.017**
AUS$30,000–59,999	1.090 (0.439–2.709)	0.853
AUS$60,000–89,999	2.624 (0.966–7.133)	0.059
AUS$90,000–119,999	2.202 (0.684–7.082)	0.186
AUS$120,000–149,999	4.696 (1.169–18.863)	0.029
≥ AUS$150,000	5.013 (1.337–18.795)	0.017
**Subjective health status (Very good, good)**		**0.006**
Fair, bad, very bad	0.453 (0.258–0.798)	0.006

#### Hong Kong

In Hong Kong, 59% of respondents had higher trust in foreigners. The regression model included one variable; sex (see Table 15). Females were almost 60% less likely to have higher trust in foreigners than males (OR 0.40, 95% CI 0.29–0.56).

**Table 15:** Regression model for trust in foreigners in Hong Kong.

Predictor	OR (95% CI)	P
**Sex (Male)**		**<0.001**
Female	0.403 (0.290–0.560)	<0.001

#### Japan

In Japan, only 28% of respondents had higher trust in foreigners, and thus the regression model is based on lower trust (72% of respondents). The regression model included three independent associations; age, marital status and subjective health status (see Table 16). Respondents aged 30–39 years were 60% more likely have lower trust in foreigners than people aged 60 years or over (OR 1.60, 95% CI 1.01–2.53). Respondents who reported never being married were almost 50% less likely to have lower trust in foreigners than people who are married or cohabiting (OR 0.55, 95% CI 0.34–0.89). Respondents with poor perceived health were almost 80% more likely to report lower trust in foreigners (OR 1.77, 95% CI 1.32–2.37).

**Table 16:** Regression model for distrust in foreigners in Japan.

Predictor	OR (95% CI)	P
**Age (≥60)**		**0.016**
20–29	1.386 (0.791–2.429)	0.254
30–39	1.597 (1.007–2.531)	0.047
40–49	0.777 (0.503–1.200)	0.256
50–59	0.779 (0.517–1.175)	0.234
**Marital status (Married/cohabitating)**		**0.008**
Separated/divorced/widowed	1.580 (0.956–2.611)	0.074
Never married	0.548 (0.336–0.891)	0.015
**Subjective health status (Very good, good)**		**<0.001**
Fair, bad, very bad	1.767 (1.316–2.371)	<0.001

#### South Korea

In South Korea, only 24% of respondents had higher trust in foreigners. The regression model, based on lower trust, included one variable; income (see Table 17). Respondents with an income in the middle third were 70% more likely to have lower trust in foreigners compared to those in the lowest income group (OR 1.70, 95% CI 1.04–2.77).

**Table 17:** Regression model for distrust in foreigners in South Korea.

Predictor	OR (95% CI)	P
**Monthly household income (Lower third)**		**0.003**
Middle third	1.702 (1.044–2.774)	0.033
Upper third	0.727 (0.510–1.035)	0.077

#### Taiwan

There was no statistically significant model for trust in foreigners in Taiwan.

#### Thailand

In Thailand, only 21% of respondents had higher trust in foreigners. The regression model (based on lower trust) included five variables; sex, age, marital status, work status and income (see Table 18). Females were more than 50% more likely to have lower trust in foreigners than males (OR 1.54, 95% CI 1.14–2.08). Respondents aged 40–49 years were 45% less likely to have lower trust in foreigners (OR 0.55, 95% CI 0.30–0.99) and respondents aged under 20 years were almost 50% less likely to have lower trust in foreigners (OR 0.42, 95% CI 0.18–1.00) than those aged 60 years or above. Compared to married/cohabitating respondents, those separated, divorced or widowed were over 50% less likely to have lower trust in foreigners (OR 0.49, 95% CI 0.30–0.78). A similar, although only marginally significant result, was obtained for never married respondents. Respondents who work without pay, students or unemployed were over 50% more likely to have lower trust in foreigners (OR 1.51, 95% CI 1.04–2.20). Respondents with an income in the upper third were 40% less likely to have lower trust in foreigners compared to people in the lowest income group (OR 0.60, 95% CI 0.41–0.87).

**Table 18:** Regression model for distrust in foreigners in Thailand.

Predictor	OR (95% CI)	P
**Sex (Male)**		**0.005**
Female	1.538 (1.139–2.077)	0.005
**Age (≥60)**		**0.020**
<20	0.422 (0.178–1.000)	0.050
20–29	0.575 (0.313–1.059)	0.076
30–39	1.012 (0.549–1.865)	0.970
40–49	0.548 (0.304–0.988)	0.045
50–59	0.900 (0.476–1.698)	0.744
**Marital status (Married/cohabitating)**		**0.004**
Separated/divorced/widowed	0.487 (0.304–0.780)	0.003
Never married	0.691 (0.470–1.017)	0.061
**Work status (Full time/self-employed)**		**0.030**
Part time	2.317 (0.879–6.108)	0.089
Working without pay/student/unemployed/other	1.511 (1.039–2.196)	0.031
Retired/pensioner	0.445 (0.166–1.192)	0.107
Household duties	1.133 (0.559–2.298)	0.729
**Monthly household income (Lower third)**		**0.027**
Middle third	0.727 (0.493–1.073)	0.109
Upper third	0.598 (0.411–0.871)	0.007

### Trust in People of a Different Religion

#### Australia

In Australia, 84% of respondents had higher trust in people of a different religion. The regression model included four independent associations; age, marital status, work status and income (see Table 19). Respondents aged under 20 years were 99% less likely to have higher trust in people of a different religion (OR 0.01, 95% CI 0.00–0.05) and respondents aged 20–29 years were 85% less likely to trust (OR 0.15, 95% CI 0.04–0.55), when compared to people aged 60 years or above. Never married respondents were 5.6 times more likely to have higher trust in people of a different religion compared to those who are married or cohabitating (OR 5.64, 95% CI 2.42–13.16). Compared to respondents in full-time employment, people in part-time employment were over 3 times more likely to have higher trust in people of a different religion (OR 3.35, 95% CI 1.38–8.14) and people reporting household duties were over 6 times more likely to trust (OR 6.40, 95% CI 1.63–25.24). Respondents in the highest income group ($150,000 and over) were 8.5 times more likely to have higher trust in people of a different religion when compared to people with incomes less than $30,000 (OR 8.56, 95% CI 1.78–41.24).

**Table 19:** Regression model for trust in people with different religion in Australia.

Predictor	OR (95% CI)	P
**Age (≥60)**		**<0.001**
<20	0.009 (0.002–0.053)	<0.001
20–29	0.151 (0.042–0.546)	0.004
30–39	0.416 (0.121–1.424)	0.162
40–49	0.401 (0.118–1.363)	0.143
50–59	0.529 (0.160–1.757)	0.299
**Marital status (Married/cohabitating)**		**<0.001**
Separated/divorced/widowed	1.238 (0.525–2.919)	0.626
Never married	5.643 (2.420–13.160)	<0.001
**Work status (Full time/self-employed)**		**0.010**
Part time	3.352 (1.380–8.142)	0.008
Working without pay/student/unemployed/other	1.565 (0.610–4.016)	0.352
Retired/pensioner	2.402 (0.668–8.636)	0.180
Household duties	6.404 (1.625–25.236)	0.008
**Monthly household income (<AUS$30,000)**		**0.036**
AUS$30,000–59,999	1.134 (0.422–3.047)	0.804
AUS$60,000–89,999	2.008 (0.724–5.573)	0.181
AUS$90,000–119,999	2.210 (0.670–7.286)	0.193
AUS$120,000–149,999	2.953 (0.803–10.858)	0.103
≥ AUS$150,000	8.555 (1.775–41.238)	0.007

#### Hong Kong

In Hong Kong, 65% of respondents had higher trust in people of a different religion. The regression model includes three variables; sex, marital status and income (see Table 20). Females were approximately 40% less likely to have higher trust in people of a different religion compared to men (OR 0.59, 95% CI 0.41–0.85). Separated, divorced or widowed people were over 5.5 times more likely to have high trust than married people (OR 5.69, 95% CI 2.68–12.06) and people who reported never having been married were almost 3 times more likely to have high trust in people of a different religion (OR 2.84, 95% CI 1.91–4.22). Respondents in the middle and highest income groups were over twice as likely to have higher trust than people in the lowest income group (OR 2.31, 95% CI 1.49–3.57; and OR 2.07, 95% CI 1.32–3.23 respectively).

**Table 20:** Regression model for trust in people with different religion in Hong Kong.

Predictor	OR (95% CI)	P
**Sex (Male)**		**0.005**
Female	0.587 (0.406–0.850)	0.005
**Marital status (Married/cohabitating)**		**<0.001**
Separated/divorced/widowed	5.686 (2.681–12.058)	<0.001
Never married	2.841 (1.912–4.223)	<0.001
**Monthly household income (Lower third)**		**<0.001**
Middle third	2.309 (1.492–3.572)	<0.001
Upper third	2.065 (1.319–3.231)	0.002

#### Japan

In Japan, only 30% of respondents had higher trust in people of a different religion. The regression model (related to lower trust) included two variables; age and subjective health status (see Table 21). Respondents aged 20–29 and 30–39 were approximately 3 times more likely to have lower trust in people of a different religion when compared to people aged 60 years or older (OR 2.71, 95% CI 1.70–4.34; and OR 3.20, 95% CI 2.02–5.06 respectively). People with poor self-rated health were over 60% more likely to have lower trust in people of a different religion when compared to people with better self-rated health (OR 1.64, 95% CI 1.22–2.21).

**Table 21:** Regression model for distrust in people with different religion in Japan.

Predictor	OR (95% CI)	P
**Age (≥60)**		**<0.001**
20–29	2.714 (1.696–4.344)	<0.001
30–39	3.195 (2.017–5.059)	<0.001
40–49	1.259 (0.832–1.905)	0.276
50–59	1.391 (0.939–2.061)	0.100
**Subjective health status ((Very) Good)**		**0.001**
Fair, bad, very bad	1.642 (1.220–2.210)	0.001

#### South Korea

In South Korea, only 38% of respondents had higher trust in people of a different religion. The regression model (for lower trust) included two variables; age and subjective health status (see Table 22). Respondents aged 20–29 and 30–39 were approximately twice as likely to have lower trust in people of a different religion when compared to people aged 60 years or older (OR 1.91, 95% CI 1.18–3.10; and OR 1.74, 95% CI 1.09–2.78 respectively). People with poor self-rated health were almost 40% more likely to have lower trust in people of a different religion when compared to people with better self-rated health (OR 1.39, 95% CI 1.03–1.89).

**Table 22:** Regression model for distrust in people with different religion in South Korea.

Predictor	OR (95% CI)	P
**Age (≥60)**		**0.026**
<20	1.055 (0.453–2.460)	0.901
20–29	1.914 (1.180–3.103)	0.008
30–39	1.741 (1.092–2.777)	0.020
40–49	1.401 (0.892–2.201)	0.143
50–59	0.992 (0.608–1.617)	0.974
**Subjective health status (Very good, good)**		**0.034**
Fair, bad, very bad	1.391 (1.025–1.887)	0.034

#### Taiwan

In Taiwan, 62% of respondents had higher trust in people of a different religion. The regression model included two variables; marital status and work status (see Table 23). Respondents who reported being separated, divorced or widowed were 2.7 times more likely to have higher trust in people of a different religion than married/cohabiting respondents (OR 2.74, 95% CI 1.64–4.57). Compared to individuals in full-time employment, pensioners/retirees were over 90% more likely to have higher trust in people of a different religion (OR 1.95, 95% CI 1.26–3.01).

**Table 23:** Regression model for trust in people with different religion in Taiwan.

Predictor	OR (95% CI)	P
**Marital status (Married/cohabitating)**		**0.001**
Separated/divorced/widowed	2.737 (1.640–4.569)	<0.001
Never married	1.095 (0.815–1.471)	0.547
**Work status (Full time/self-employed)**		**0.064**
Part time	1.123 (0.671–1.881)	0.658
Working without pay/student/unemployed/other	1.071 (0.658–1.743)	0.782
Retired/pensioner	1.945 (1.256–3.012)	0.003
Household duties	1.130 (0.725–1.762)	0.589

#### Thailand

In Thailand, only 23% of respondent had higher trust in people of a different religion. The regression model (based on lower trust) included five variables; sex, age, work status, marital status and income (see Table 24). Females were approximately 40% more likely to have lower trust in people of a different religion than males (OR 1.39, 95% CI 1.05–1.86). Compared to respondents aged 60 years or over, people under 20 years were approximately 70% less likely to have lower trust in people of a different religion (OR 0.33, 95% CI 0.14–0.75). Compared to married/cohabitating respondents, individuals who were separated, divorced or widowed approximately 50% less likely to have lower trust in people of a different religion (OR 0.49, 95% CI 0.31–0.77). Respondents who work without pay or are unemployed/student were more than twice as likely to have lower trust in people of a different religion (OR 2.25, 95% CI 1.55–3.27). Respondents with an income in the middle third were over 30%, and respondents with an income in the upper third were 45% less likely to have lower trust in people with a different religion compared to respondents in the lowest income group (OR 0.69, 95% CI 0.47–0.99; and OR 0.55, 95% CI 0.38–0.79).

**Table 24:** Regression model for distrust in people with different religion in Thailand.

Predictor	OR (95% CI)	P
**Sex (Male)**		**0.024**
Female	1.394 (1.045–1.860)	0.024
**Age (≥60)**		**0.036**
<20	0.329 (0.144–0.753)	0.009
20–29	0.699 (0.391–1.251)	0.228
30–39	1.012 (0.573–1.785)	0.968
40–49	0.765 (0.436–1.341)	0.349
50–59	1.131 (0.615–2.081)	0.692
**Marital status (Married/cohabitating)**		**0.007**
Separated/divorced/widowed	0.489 (0.310–0.773)	0.002
Never married	0.801 (0.550–1.167)	0.248
**Work status (Full time/self-employed)**		**<0.001**
Part time	2.068 (0.888–4.819)	0.092
Working without pay/student/unemployed/other	2.249 (1.545–3.273)	<0.001
Retired/pensioner	2.165 (0.655–7.160)	0.205
Household duties	1.919 (0.934–3.942)	0.076
**Monthly household income (Lower third)**		**0.006**
Middle third	0.685 (0.470–0.998)	0.049
Upper third	0.548 (0.380–0.790)	0.001

By way of summarising the main findings from the preceding regression models, Table 25 presents the main factors predicting low trust: age, income, gender and health status. Younger people have lower trust in all groups within Australia and Japan and lower trust in neighbours and people with a different religion in South Korea. However, older people had lower trust in foreigners and people of a different religion in Thailand. The findings for income differed by country. In all countries except South Korea, being on a low income was associated with lower trust, predicting low trust in neighbours in Australia, Japan and Taiwan and low trust in people with a different religion in Australia, Hong Kong and Thailand. However, being on a higher income in South Korea was related to lower trust in strangers and foreigners. The findings were conflicting for gender. Generally, females had lower trust than males in Thailand and Hong Kong, although in Australia, males had lower trust than females in strangers, whereas females had lower trust than males in foreigners. In Hong Kong and Thailand, females had lower trust than males in foreigners and people with a different religion. Gender was not associated with trust in Japan, South Korea or Taiwan. Finally, having either poor subjective health or a chronic condition was associated with lower trust in all countries except Hong Kong and Taiwan. Indeed, having poor subjective health or a chronic condition was associated with lower trust in all four groups in Japan and with three of the four in South Korea. In addition, poor subjective health was associated with lower trust in neighbours in Australia, Japan, South Korea and Thailand.

**Table 25:** Summary of predictors of low trust.

	*Low trust in* *neighbours*	*Low trust in* *strangers*	*Low trust in foreigners*	*Low trust in people with a* *different religion*
*AU*	Low income; Full-time employment;Younger age; Poorsubjective health	Younger age;Males	Low income; Full-time employment;Younger age; Females; Poorsubjective health	Low income; Full-time employment;Married/cohabiting;Younger age
*HK*	No model	Females; Full timeemployment	Females	Females; Low income;Married/cohabiting
*JPN*	Low income; Separated/divorced/widowed; Younger age; Poorsubjective health	Younger age; Poorsubjective health	Younger age; Married/cohabiting;Poor subjective health	Younger age; Poorsubjective health
*SK*	Younger age; Poor subjectivehealth	Higher income;Chronic condition	Higher income	Younger age; Poorsubjective health
*TWN*	Low income; Never married;Part-timeemployment	Low income	No model	Married/cohabiting;Pensioners or retired
*THL*	Poor subjective health	50–59years	Low income; Married/cohabiting;No pay/students/unemployed; Females;Higher age	Low income; Married/cohabiting;No pay/students/unemployed;Females; Higher age

## Discussion

The descriptive data presented in this paper shows very high levels of trust in the family across all countries, although trust in the other groups was variable. There was generally high trust in neighbours, although this varied from 92% in Thailand to 75% in Hong Kong. Trust in people with a different religion was markedly varied, with only 23% of respondents indicating higher trust in Thailand, whereas over 60% in Taiwan and Hong Kong and over 80% in Australia indicated higher trust in this group. Trust in foreigners presented a similar pattern, with only 21% of respondents in Thailand indicating higher trust whereas this was 85% in Australia. The group with the lowest levels of trust was ‘strangers’, with levels of higher trust being 10% in Thailand, 17% in Hong Kong, South Korea and Taiwan and up to 57% in Australia.

The almost complete trust in the family is seen across all countries in this paper, thereby questioning Fukuyama’s suggestion that trust in the family will be higher in Confucianist societies. It seems that trust in the family is blind to cultural context, and happens irrespective of nation state or cultural norms. Govier examines why trust exists within families, even when any number of broken promises or even abuse might have happened, and she concludes her analysis by stating “For all its flaws, for all its risks, our family is our first human circle” (p.86). Therefore, in comparing and contrasting the countries in this paper, we need to focus on what Fukuyama calls ‘spontaneous sociability’ (p.27), which the variety of groups or inter-relationships outside of the family but not deliberately set up by governments. These can be neighbours, community groups or chance meetings with strangers in the street. To what extent is there spontaneous sociability within the countries studies in this paper.

The general findings of lower trust in ‘unknown’ people such as strangers, foreigners and people with a different religion is not unexpected – one would expect this. However, the difference in levels of trust between countries provides areas for further research and possibly policy development to increase both trust and trustworthiness, especially of foreigners and people of a different religion because the levels of distrust may lead to further discrimination and social exclusion of these groups, perpetuating a cycle of distrust. Indeed research in the US identified that one of the strongest factors associated with low levels of trust is belonging to a group that historically felt discriminated against [38]. The policy response then, is to address real or felt discrimination as a means of mitigating low levels of trust in this US subpopulation.

Although Fukuyama argued that low trust societies, such as ones based on Confucianist ideals, have high trust within the family, our data show that all countries have high trust within the family. For example, Fukuyama [6] said “a strong family system can be seen as an essentially defensive mechanism against a hostile and capricious environment” (p.88). Whilst this may well be the case for Thailand, Japan and South Korea, which have high trust in family but lower trust in other groups, it does not explain Australia, or to a large extent, Hong Kong and Taiwan, which also have high trust in the family but, to differing levels, high trust in a number of groups outside the family. The application of Fukuyama’s thesis [6] to these countries would render Australia, Japan and South Korea as having higher trust, with Hong Kong, Taiwan and possibly Thailand as having lower trust. Whilst our data certainly show Australia as having the highest levels of trust, we categorise Japan and South Korea as having lower trust outside the family than either Hong Kong or Taiwan, which goes against Fukuyama’s Confucianist interpretation. Respondents in both Hong Kong and Taiwan have higher trust in people of a different religion and foreigners than respondents in Japan or Taiwan.

The notion of high trust and low trust societies was interrogated in more detail in Table 2, which provides a more complex picture of trust. Developing a more complex conceptualisation of trust than the rather rigid high/low trust societies has also been proposed by others [19], [39]. Out of the four individuals or groups (neighbours, foreigners, strangers and people with a different religion), Australia and Hong Kong had statistically significantly higher levels of trust in 3/4, Japan and Taiwan in 2/4, South Korea in 1/4, and Thailand in 0/4. This suggests that Australia and Hong Kong may be categorised as ‘high trust societies’ (although the z-scores were much higher in Australia, indicating higher trust), Japan and Taiwan as ‘medium trust societies’ and South Korea and Thailand as ‘low trust societies’. Although our tripartite analysis of overall levels of trust does not fit with Fukuyama’s dichotomisation, it nevertheless provides some corroboration and refutation. Australia has the highest levels of trust in both Table 1 and Table 2, fitting in with Fukuyama’s ‘high trust society’. However, the analysis in Table 2 shows Hong Kong with a higher level of trust than Taiwan, whereas we categorised them both as ‘medium level trust’ from Table 1. Table 2 shows Hong Kong as a ‘high trust society’ whereas Fukuyama’s analysis would regard it as having lower trust than either Japan or South Korea. Indeed, our data show that South Korea has lower levels of trust than either of the countries based on Confucianism (Hong Kong and Taiwan).

Research demonstrates that trust in strangers and acquaintances varies across the globe, For example, research analysing data across 60 nations identifies high trust countries characterised as being of ethnic homogeneity, Protestant religious traditions, good government, wealth (gross domestic product per capita), and income equality [40]. This variability too is evident in our data. However, further to the categorization of low, medium and high trust societies, our data demonstrate subpopulations where trust *within* each society is low. As noted, Fukuyama did not theorise differences in trust across subpopulations within high/low trust societies. Previous research identifies individual and country-related characteristics found to effect social trust [41]. Our data permit an investigation of the specific subpopulation within each of the low, medium and high trusting societies. Firstly, we can identify low trusting subpopulations in ‘high’ trust societies. For example, in Australia, lower trust was found to be consistent for individuals of younger age, low income, poor self-rated health and full-time employment (to be categorised as consistent, the (socio) demographic must have been predictive for ≥2 of the four groups of individuals). In contrast, females were the only subpopulation found to consistently have low levels of trust in Hong Kong. Secondly, we can identify low trusting subpopulations in ‘medium’ trust societies. For example, in Japan, individuals of younger age and poor self-rated health consistently had lower levels of trust. The results for Taiwan differed, with the lowest levels of trust identified in the low-income population group. Finally, we can identify low trusting subpopulations in ‘low’ trust societies. For example, in South Korea, individuals of higher income, poor self-rated health and younger age were found to be the least trusting subpopulation. The results from Thailand suggest that individuals of higher age, low-income, married or cohabitating, or are students/unemployed are the least trusting population groups.

The above categorisations demonstrate the variability within countries. Of interest are the noted trends of subpopulations with low trust across the countries: generally people on low incomes, younger people and people with poor self-rated health. People on low incomes have been found to have lower inter-personal trust in a number of social relationships [42], [43], [44], with the suggestion that people who are more ‘successful’ in life (based on education and income) are more likely to have higher trust [42] The findings of lower trust in younger groups has been found in a cross-sectional study in the US [45] in addition to an experimental study in Europe [46]. In both of these studies, generalised trust increased with age, which was also evident across most studies in this paper. Similar to our findings on gender, men have been found to have higher levels of trust in unknown people (e.g. strangers) in the US [47] and in China [22]. Likewise, international research has also shown similar findings in relation to people with poorer self-rated health having lower levels of trust [48], [49].

Following Fukuyama, but cognisant of critiques of his work [19], the findings from this paper point to the complexity of trust both within and across cultural groups and social norms. Our findings extend Fukuyama’s thesis and open up further areas for empirical investigations within low, medium and high trust societies. Additionally, the findings permit movement towards implications as we have more explicit detail regarding the low trusting populations within each society, regards of their overall categorisation. Generalized social trust is beneficial for societies, found to be correlated with increased levels of civic engagement, lower crime rates, and greater economic growth [41] (p.61). Given the importance of trust for building social networks, social capital and a more basic sense of individual and societal wellbeing [50], [51], [52], these findings outline some areas worthy of further research and possible policy development.

Our statistical models based on more generalised forms of trust may be a good starting point for further qualitative research studies. Such studies could begin to understand and contextualize other possible factors involved in trust or distrust, and may provide a more detailed analysis to explore ‘trust in context’. Such an approach may then examine the lived experience and understanding of trust. In addition, whilst our paper rests on the default position of trust being a generally ‘good thing’, it may be the case in certain contexts where a default position of lack of trust, or at least critical reflexivity, helps to buffer social groups from oppression. As mentioned earlier, Govier [9] argues that such a position may guard against complacency.

In terms of possible policy implications of our findings, the imputation of possible policy responses needs to be interpreted within caution, since much debate has ensued in this area. At one level, we agree with Govier that when she provides a critique of whether or not to trust a stranger, “We can expect no guidebook telling us how to do this, no formula or magic rule offering infallible advice as to whether to trust a stranger at the doorstep” (p.204). This advice may also be extended to policy makers when thinking about developing policies to increase trust within societies. Indeed, after the publication of his book on Trust, Fukuyama also explored the policy options to increase social capital and trust, concluding that direct policy directives may be limited due to the social, cultural, religious and historical determinants of trust in countries [53]. Instead of direct policy, Fukuyama argued that we at least need to raise awareness of trust (or mistrust) in order for development to be enhanced. It is hoped that this paper has further ploughed this furrow.
